# Supplementary material for: Constructing the human brain metabolic connectome with MR spectroscopic imaging reveals cerebral biochemical organization
Source: Nat Commun. 2025 Dec 22;16:11344. doi: 10.1038/s41467-025-66124-w (PMC12727723; doi:10.1038/s41467-025-66124-w)
Supplement: Supplementary file 1 — Supplementary Information [file 41467_2025_66124_MOESM1_ESM.pdf]

Supplementary Figures

0.1 MetSiM Connectome Coverage

After individual ^{1}H-MRSI volume parcellations, regions with fewer than 10 ^{1}H-MRSI voxels in more than 70% of subjects were excluded, resulting in the effective MetSiM coverage shown in Supplementary Fig.1. The excluded regions were primarily located in the orbitofrontal and basotemporal areas, where ^{1}H-MRSI coverage is limited due to susceptibility artifacts and signal distortion. Additionally, the inferior cerebellum was excluded as it was not covered by the ^{1}H-MRSI field of view. Raw data is reported in Supplementary Data 1.

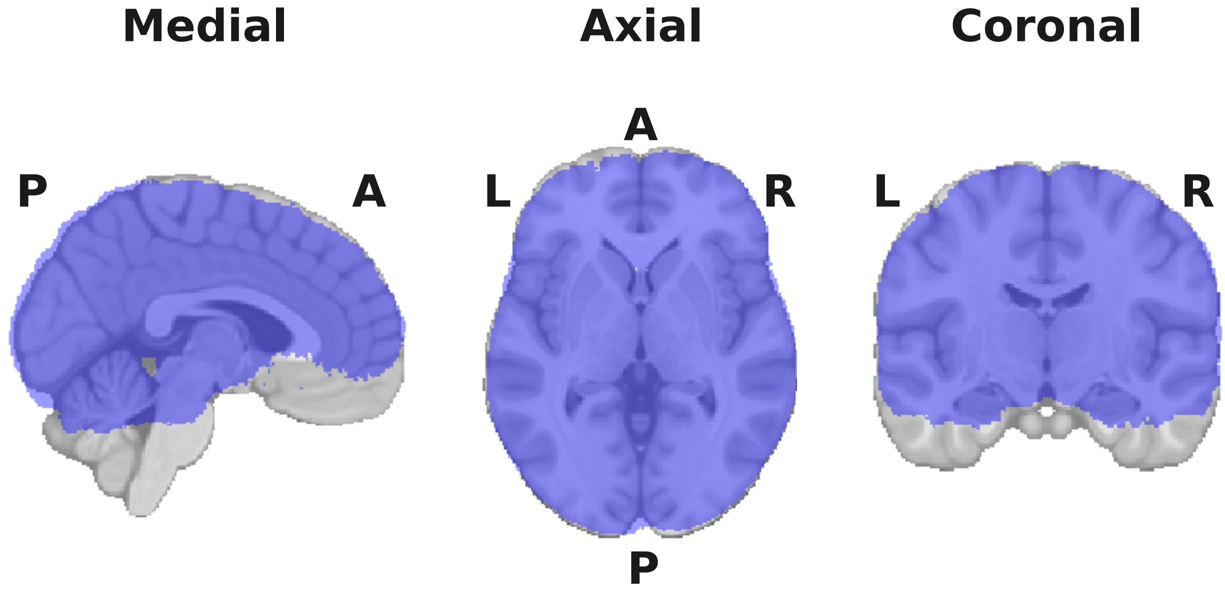

Supplementary Figure 1: **MetSiM Coverage** Effective MetSiM coverage for the Geneva sample ($N = 51$), excluding regions with limited ^{1}H-MRSI voxels (orbitofrontal, basotemporal) and the inferior cerebellum not covered by the sequence's field of view.

0.2 Reconstructed MS mode Matrix

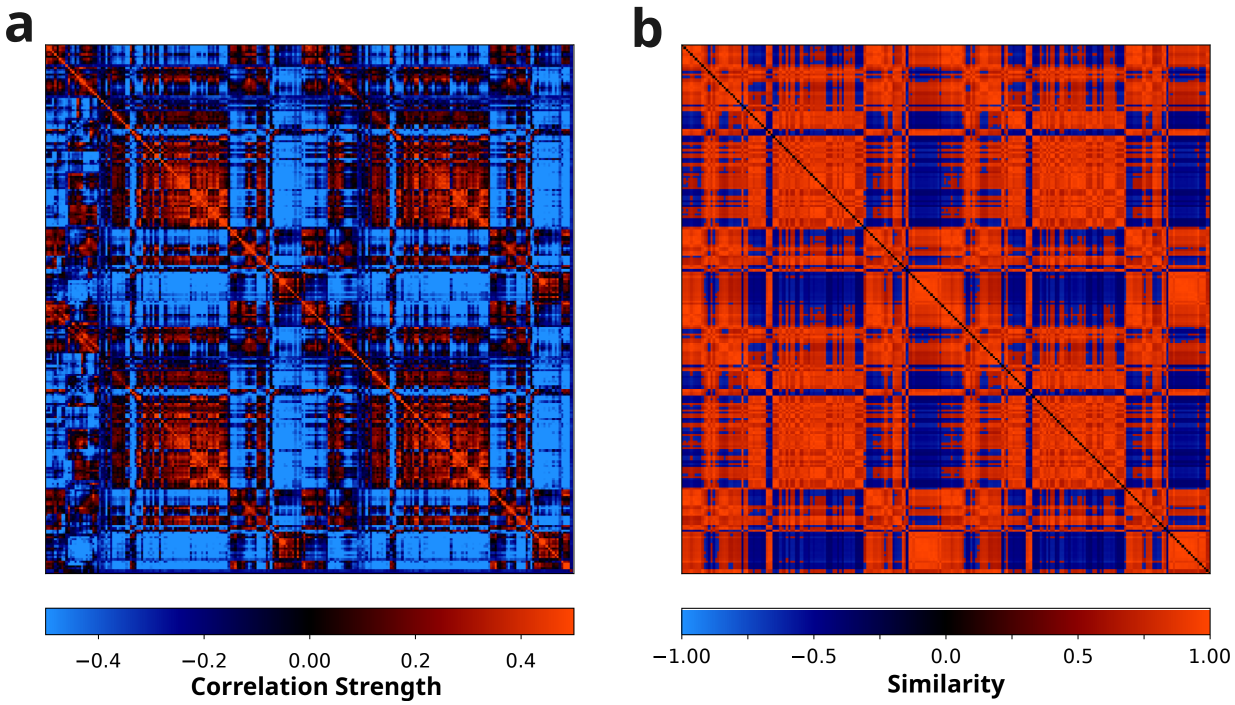

Supplementary Figure 2: **MetSiM Dimensionality Reduction** (a) Group-average MetSiM for the Geneva sample ($N = 51$). (b) Reconstructed MetSiM from PCA- and t-SNE–based dimensionality reduction of the group-average Geneva sample's nodal connectivity profiles.

0.3 Impact of Cortical Parcellation Schemes on MetSiM Construction

We evaluated the impact of different cortical parcellations, specifically Schaefer200 [1] (200 cortical parcels) and MIST197 [2](197 cortical parcels), on the construction of MetSiMs. These parcellations, derived from functional cortical atlases, were chosen to contrast the structurally based Lausanne parcellation in the Chimera LFMIHIFS-3 scheme, given their comparable number of cortical parcels. Both were integrated into the Chimera schema by substituting the Lausanne parcellation with Schaefer-200 and MIST197. The Gini coefficient, commonly used to assess inequality, was applied here as a measure of uniformity in parcel sizes. Lower Gini values indicate more uniformly sized parcels. The Schaefer-200 (0.21), MIST197 (0.22), and Lausanne (0.20) parcellations all exhibited low and comparable Gini coefficients, demonstrating their suitability for schemes requiring uniformly sized parcels. Additionally, we applied two cubic geometric parcellations to assess the influence of strictly uniform, grid-based parcels: one with 15 mm sides yielding 512 parcels and another with 10 mm sides yielding 1270 parcels. Because different parcellation schemes lead to differently shaped and differently mapped matrices, they cannot be directly compared. Hence, we directly compared their resulting MS mode maps, with the original MS mode map that resulted from the LFMIHIFS-3 parcellation scheme using the normalized mutual information (NMI), and supplemented the observed score with PermAdj. After discarding parcels with poor ^{1}H-MRSI coverage, Schaefer-200 and MIST197 retained 188 and 177 parcels, respectively, compared to 192 in the LFMIHIFS-3 atlas. All alternative atlases exhibited strong Pearson correlations and normalized mutual information (NMI) with LFMIHIFS-3: MIST197 ($r = 0.91$, NMI= 0.64), Schaefer-200 ($r = 0.74$, NMI= 0.65), and both cubic parcellations ($r = 0.99$, NMI= 0.99) (all $p < 0.05$, PermAdj), indicating their equivalence in generating MS mode maps.

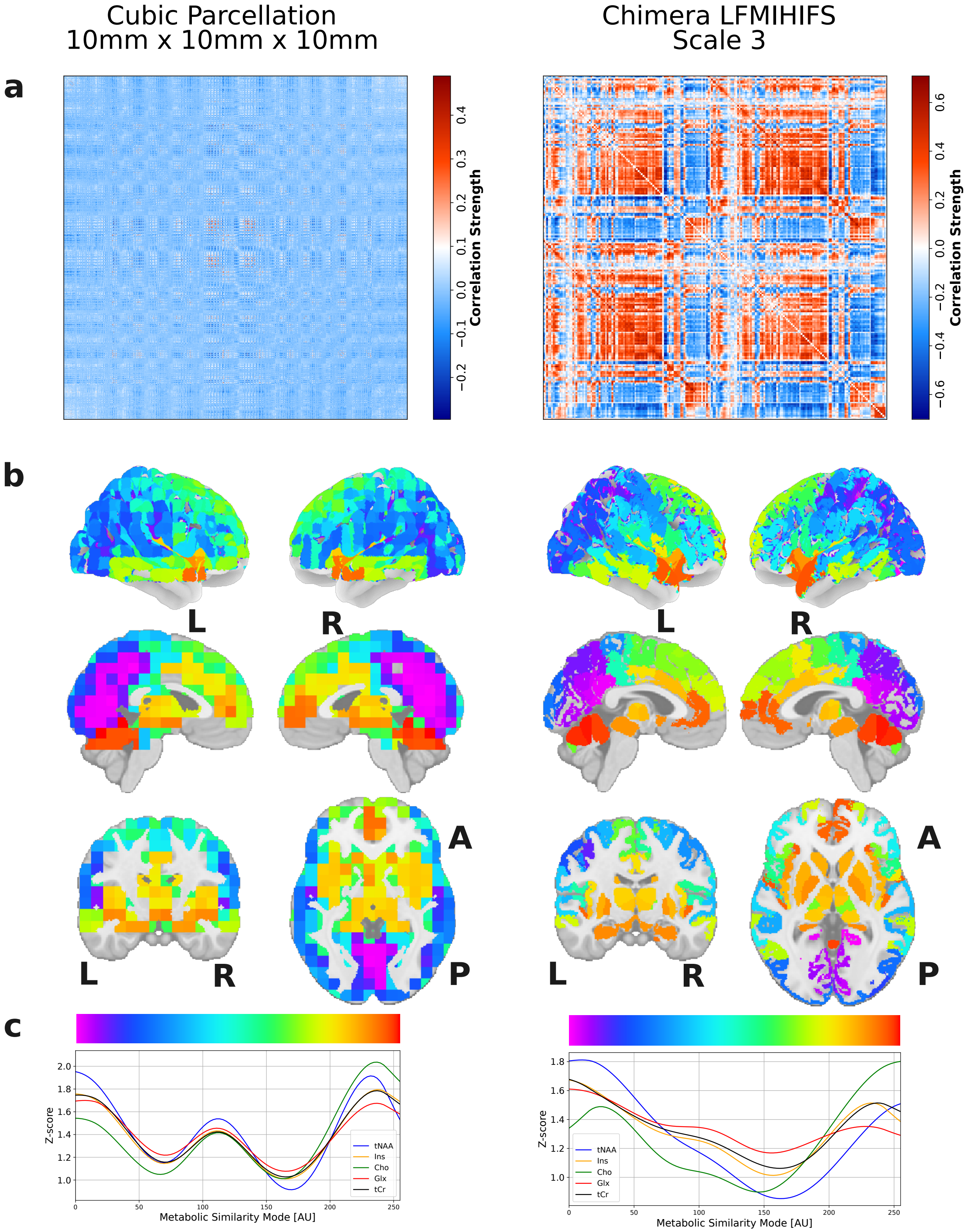

Supplementary Figure 3: **Impact of Cortical Parcellation** Left: geometric cubic (10 mm sides) parcellation with 1270 parcels; right: Chimera LFMIHIFS scale 3 parcellation with 211 parcels. **a** Metabolic similarity matrix for the Geneva Study. **b** Associated metabolic similarity index. **c** Inverse-mapped MS mode values onto metabolic profiles.

0.4 Random Geometric Models of Empirical MetSiMs

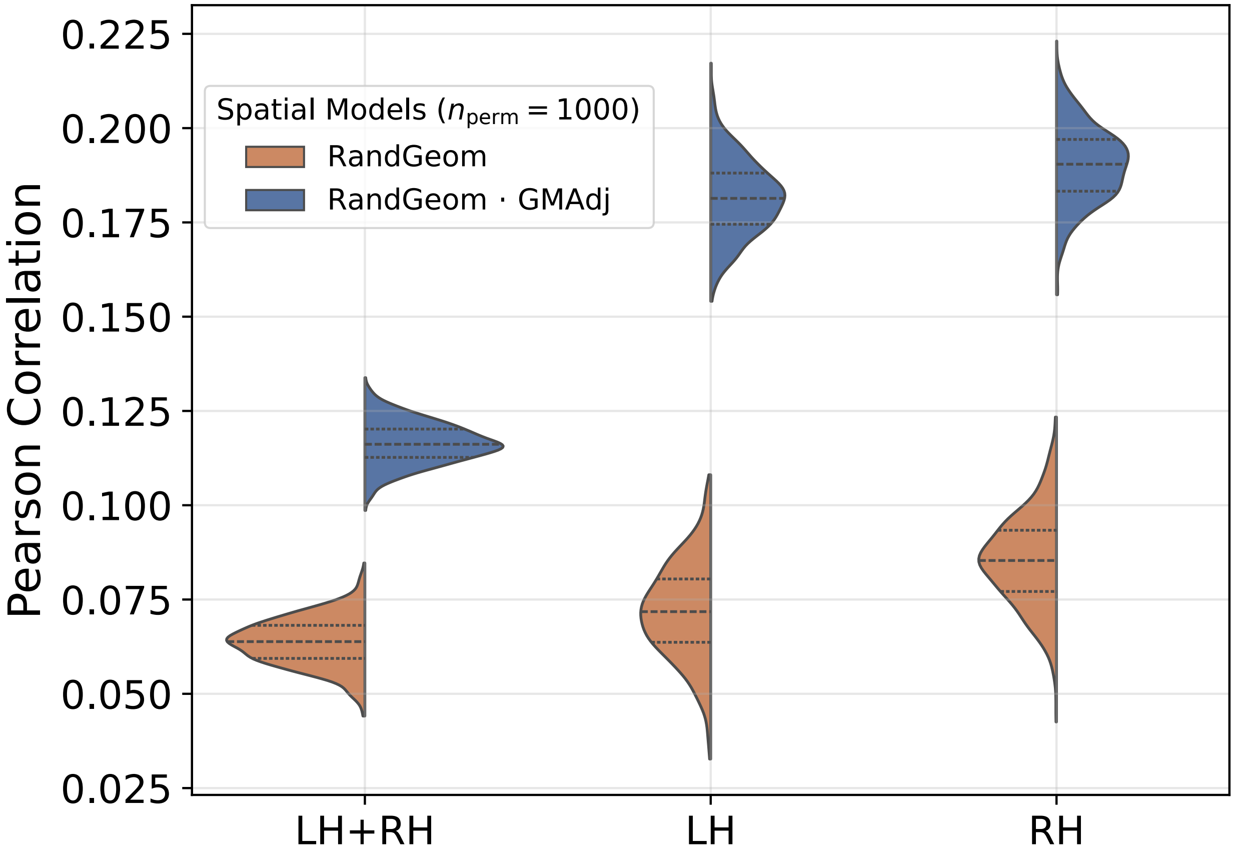

Supplementary Figure 4: **Random Geometric Models of empirical MetSiMs.** Distributions of correlations between 1000 synthetic MetSiMs generated by random geometric models (RandGeom; RandGeom·GMAdj, restricted to gray-matter-adjacent regions) and the empirical MetSiM. Results are shown for both hemispheres (LH+RH) and for single-hemisphere models with mirrored symmetry (LH or RH). Distributions are displayed as *violin* plots; widths reflect kernel density, and the interior lines mark the 25th, 50th (median), and 75th percentiles (i.e., the three main quartiles).

0.5 Rich-Club Analysis

Rich-club coefficients were computed for the MetSiM matrices of all individuals in the Geneva sample and tested against two different null distributions, each generated from 1000 random networks: one using a degree-preserving rewiring algorithm (see Methods Rich-Club Analysis), and the other using a random geometric model that mimics the spatial distribution of empirical MetSiM edges (see Methods Spatial Null Models), across edge densities ranging from 1% to 30% (see Supplementary Fig. 3a). The same analysis was applied to the group-averaged MetSiM ($N = 51$) (see Supplementary Fig. 3b). Rich-club coefficients were significant across all subjects and the averaged MetSiM.

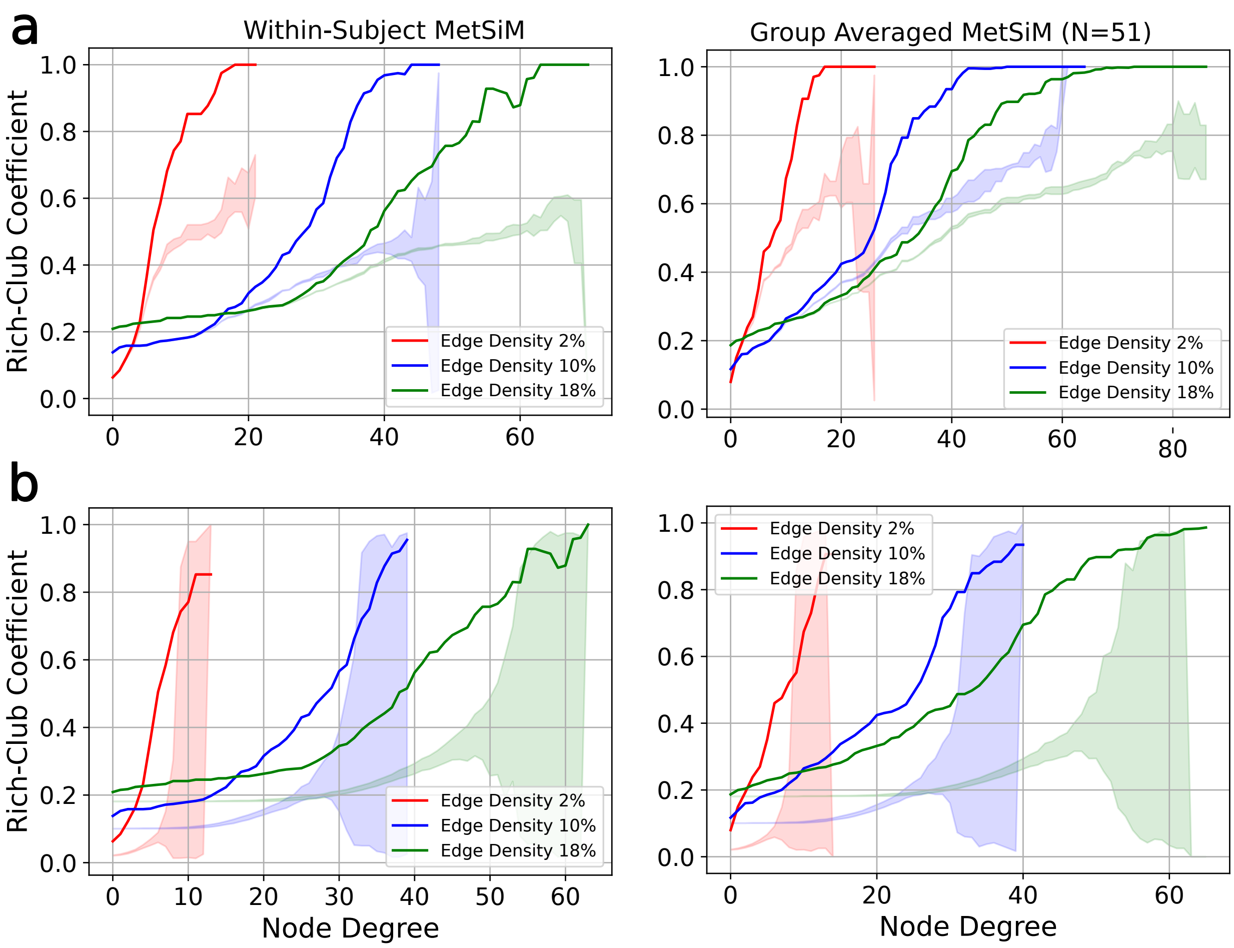

Supplementary Figure 5: **MetSiM rich–club coefficient analysis.** Rich–club coefficients for MetSiM matrices compared with (top) degree-preserving random graphs and (bottom) spatial random geometric graphs. **a**, Individual MetSiMs ($n = 51$ participants): rich–club coefficient as a function of node degree at connection densities of 2%, 10%, 18% and 40%. Solid lines show the median across participants; shaded shaded regions depict the null envelope (pointwise 0.1–99.9th percentiles) from 1000 surrogate graphs; values above this band are significant at $\alpha = 0.001$. **b**, Group-averaged MetSiM: rich–club coefficients and null envelopes at the same densities.

0.6 Principal Path Cost Function

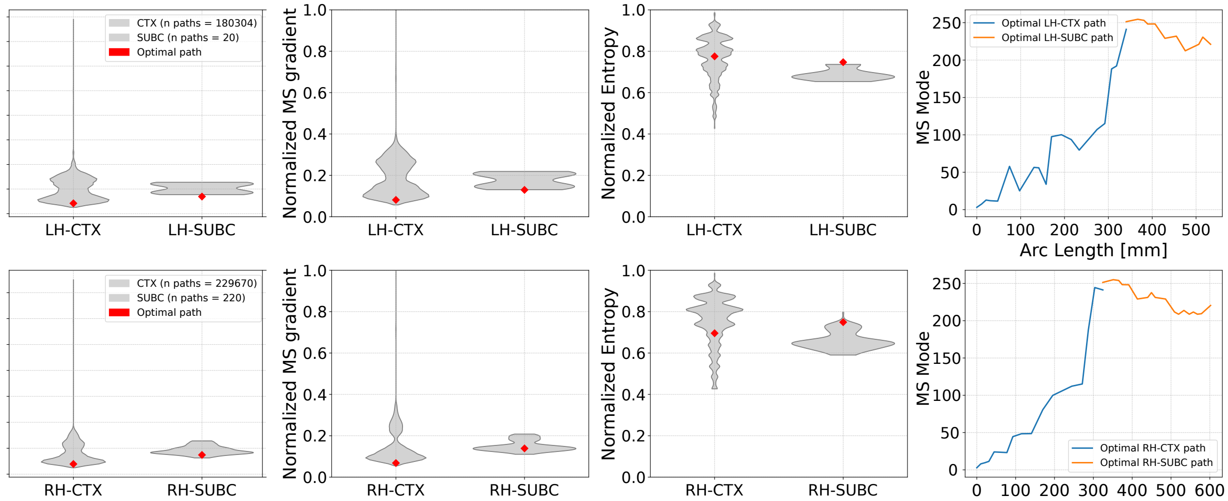

Supplementary Figure 6: **Metabolic Principal Path Cost Functions** Total cost functions, normalized MS gradient, and normalized entropy associated with path construction across the Geneva Study average MetSiM are shown for both hemispheres and for cortical and subcortical regions. Red markers indicate the optimal solutions whose MS mode values as a function of arc length are shown on the right. Shaded areas indicate the distribution of values across all suboptimal paths (neocortex: 180304 left; 229,670 right; subcortex: 20 left; 220 right).

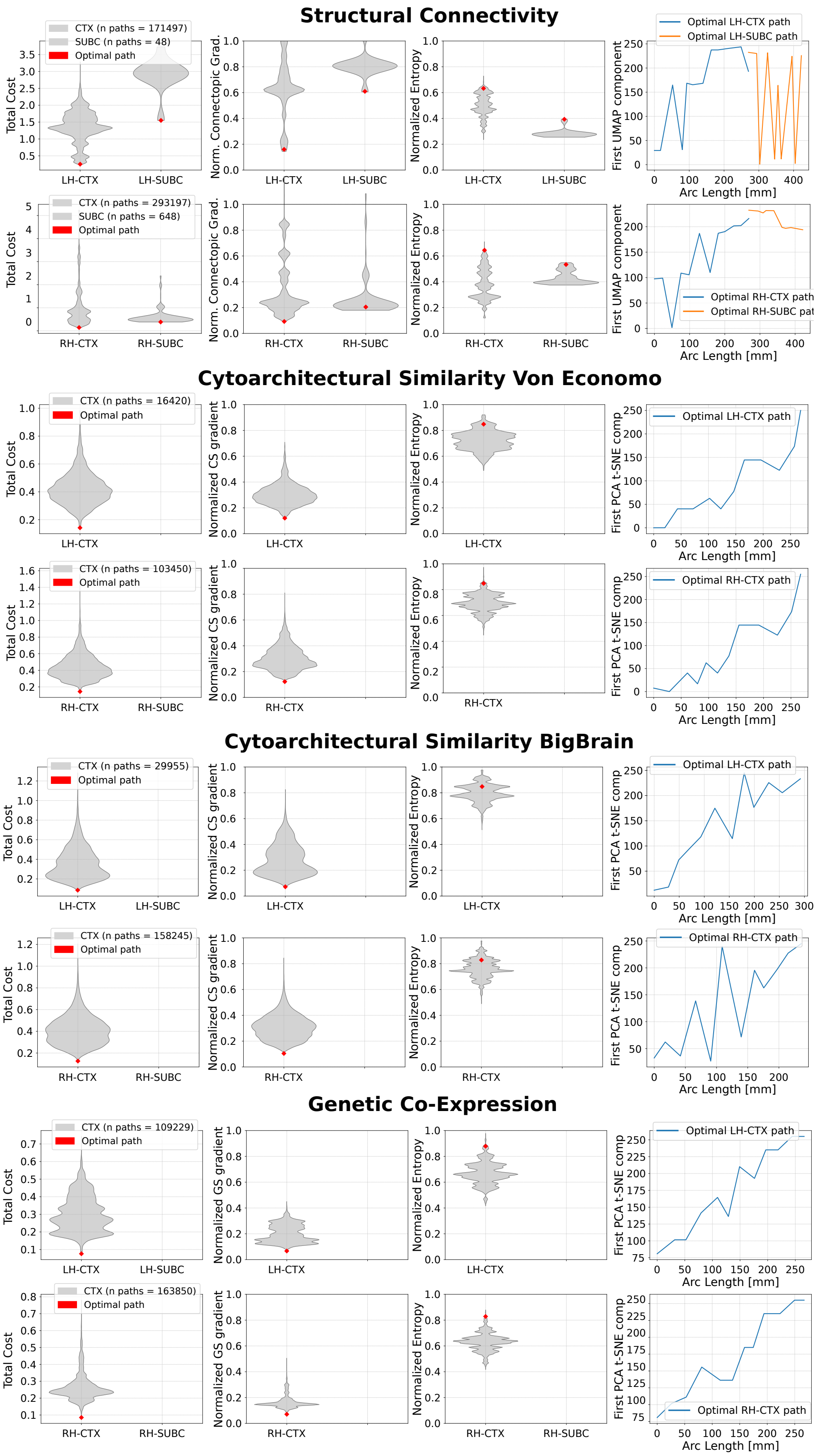

Supplementary Figure 7: **Other Modalities Principal Path Cost Functions** Total cost functions (first column), normalized network similarity gradients (first column), normalized entropy (third column) associated with the construction of paths for the Geneva Study average structural connectivity matrix, cyto.itectonic similarity matrices (Von Economo and BigBrain), and genetic co-expression matrix are

0.7 Gene Enrichment Cutoff

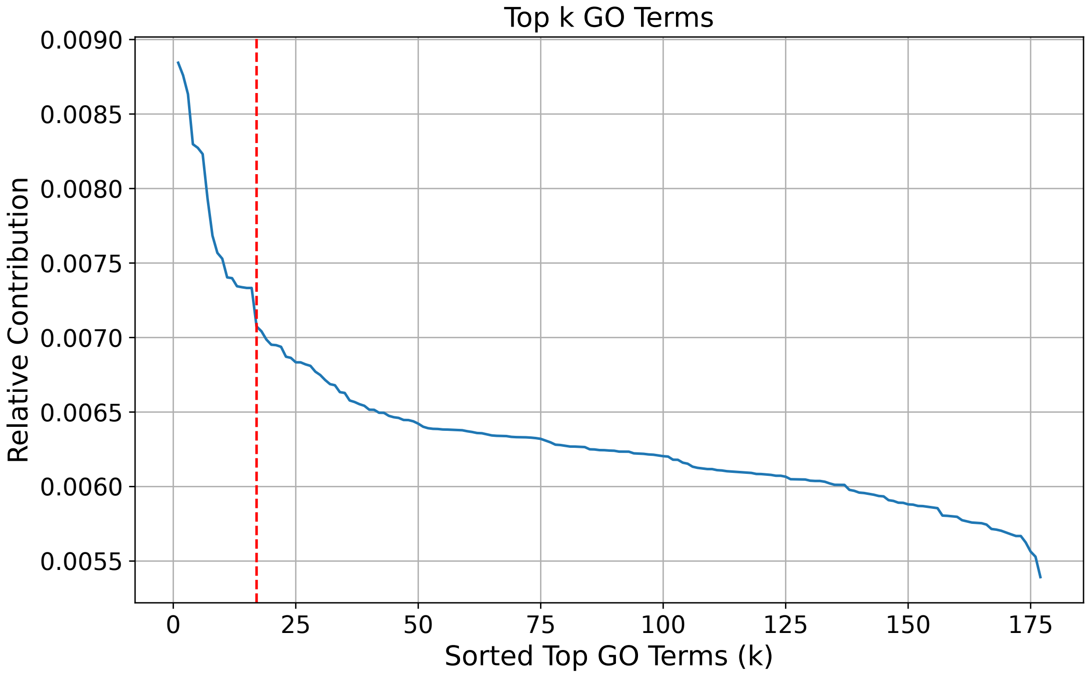

Supplementary Figure 8: **Leave-One-GO Analysis** Relative contribution was computed as the difference between the global edge-wise correlation of the group-average MetSiM (Geneva study) with the complete genetic co-expression matrix, and the correlation obtained when the genetic-coexpression matrix is imputed by the genetic expression profile corresponding to each left-out GO term. Contributions have ranked, and the elbow method identified 17 (vertical line) as the optimal number of GO terms contributing most to the overall correlation.

0.8 Sample MRSI Images

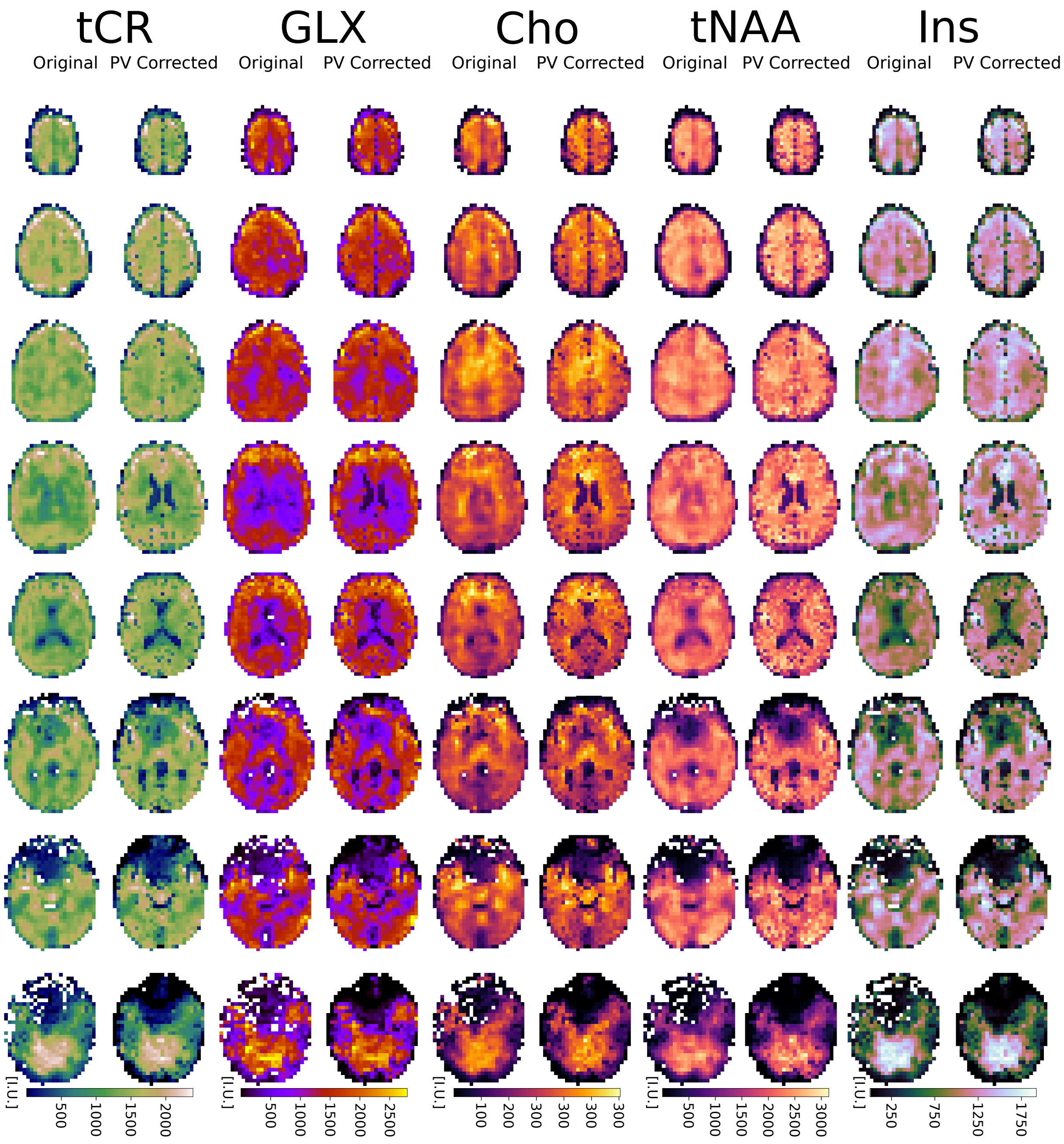

Supplementary Figure 9: **Sample MRSI Images** 8 slices (rows) of an individual MRSI sample from the Geneva study for the 5 metabolites (columns). The original raw images are shown on the left side of each column, and the corresponding partial volume–corrected images on the right, obtained using region-based voxel-wise modeling [3] with point spread function (5 mm width) deconvolution to account for MRI signal blurring.

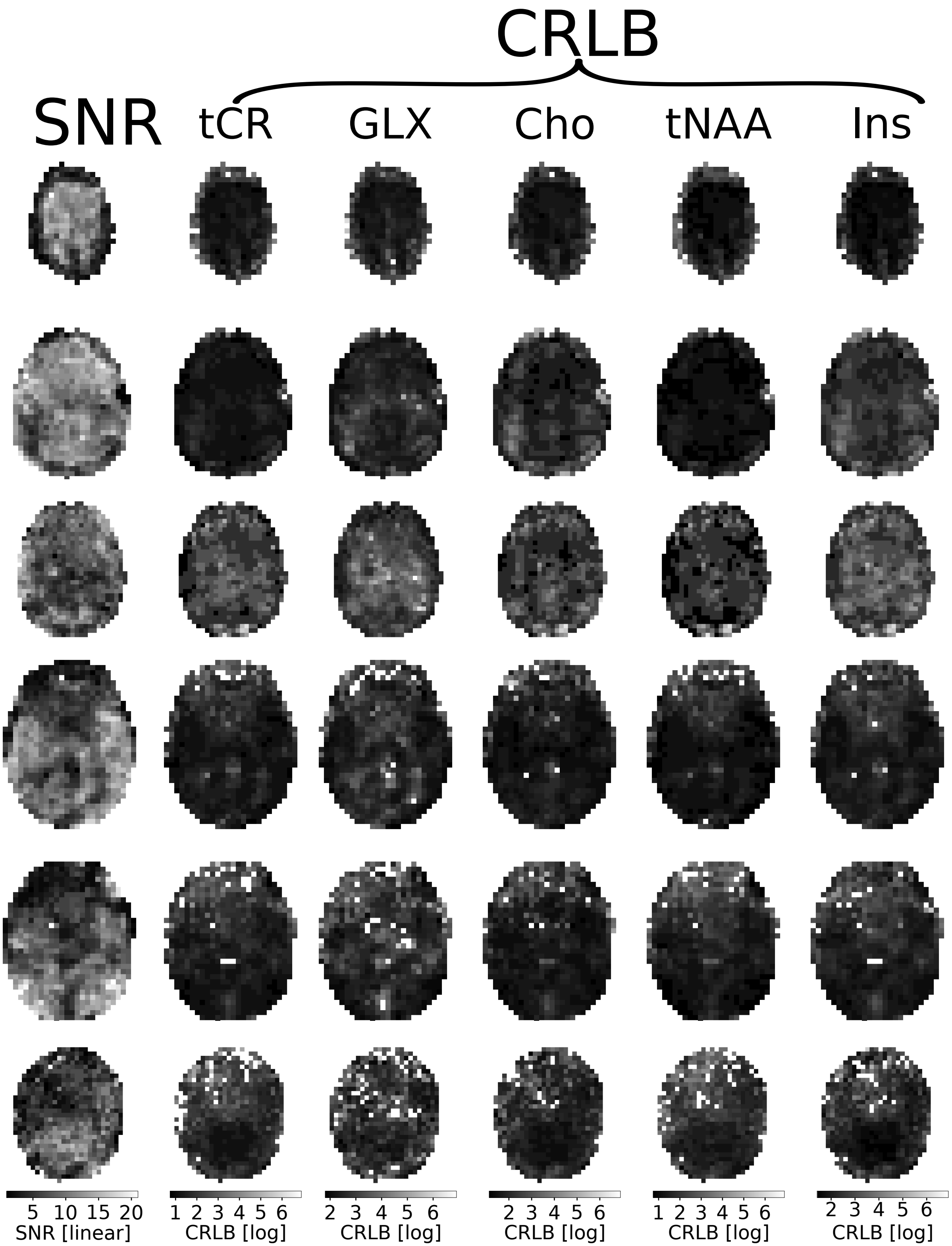

Supplementary Figure 10: **Sample MRSI SNR and CRLB images.** Axial slices (rows) from an individual MRSI dataset of the Geneva study. The first column shows the SNR map in linear scale, the remaining five columns display the CRLB (Cramér–Rao lower bound) maps for each metabolite in logarithmic scale.

0.9 Validation of MRSI-derived Metabolite Ratios against prior Literature

We validated gray-matter (GM) MRSI–derived metabolite ratios by averaging across all Geneva participants and comparing them with published values. Supplementary Fig.11 displays Cho/tNAA and the ratios tNAA/tCr, Cho/tCr, Ins/tCr, and Glx/tCr as functions of the MS mode mapped to MNI space, facilitating localization of regional trends. For Cho/tNAA, hemisphere-averaged values were $\sim$0.151 (occipital), 0.170 (parietal), and 0.205 (frontal), closely matching Maudsley et al.[4] (0.15, 0.16, 0.22) and reproducing the expected caudal-to-rostral increase. Maudsley et al. used volumetric whole-brain ^{1}H-MRSI with an interleaved water reference and automated spatial normalization (MIDAS) to generate normative metabolite maps suitable for voxel-wise group analysis. For tNAA/tCr, Maudsley et al. reported values varying around 1.4 along the same axis (1.40, 1.42, 1.44), consistent with our estimates (1.40, 1.39, 1.30). For Glx/tCr, our hemisphere-averaged values were $\sim$1.50 (occipital), 1.58 (parietal), and 1.50 (frontal), whereas Goryawala et al. [5] reported similar ranges of 1.30–1.40, 1.23–1.26, and 1.25–1.26, respectively. A thorough validation of the MRSI data used here was carried out by Celereau et al. [6], who analyzed the same datasets as in this study and also compared the WM/GM metabolite ratios with those reported in other studies, finding excellent agreement. Interested readers are referred to that work for further details.

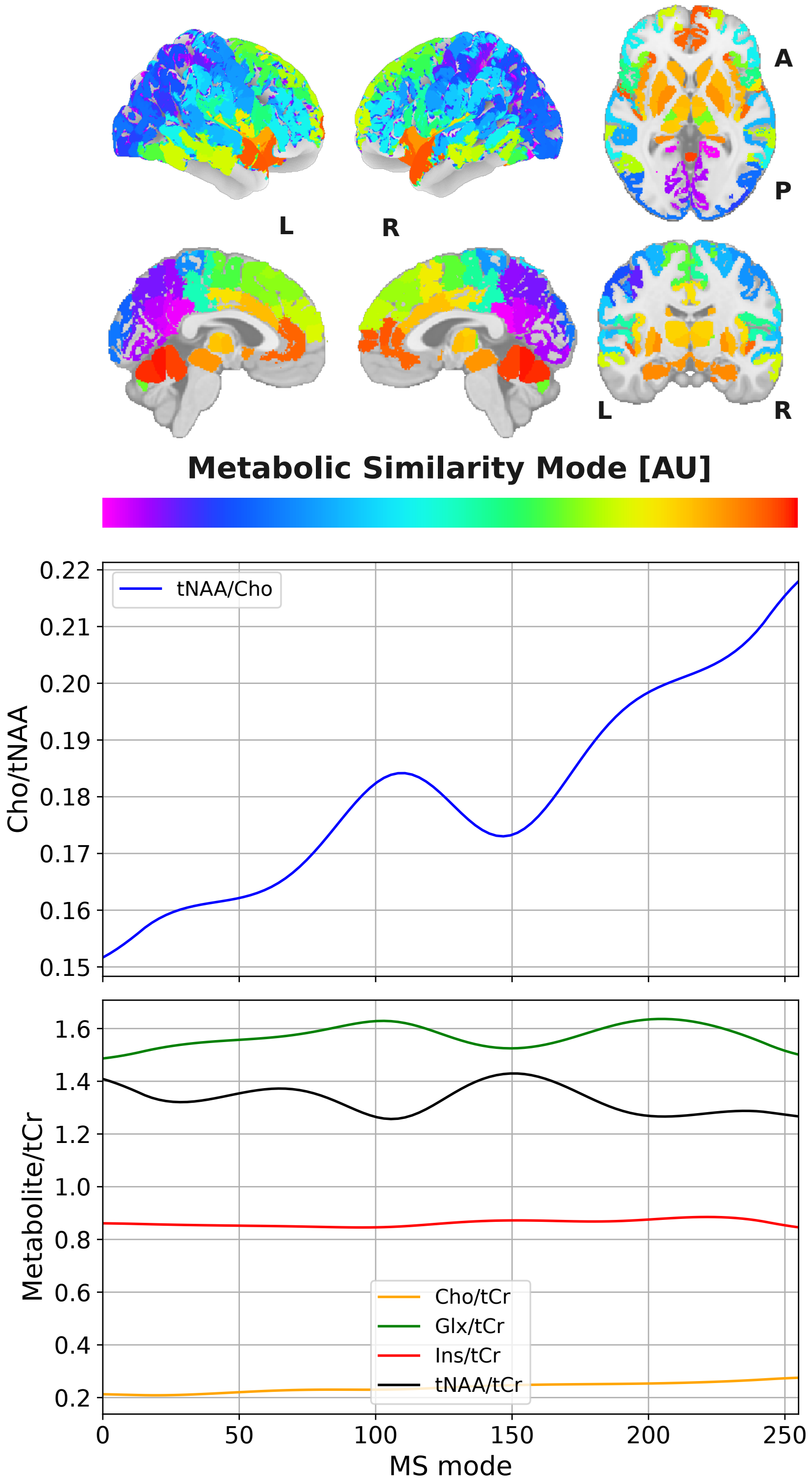

Supplementary Figure 11: **MRSI Ratios vs MS mode** Cho/tNAA plotted as a function of the MS principal mode and r atios of other metabolites to total creatine (tCr)—tNAA/tCr, Cho/tCr, Ins/tCr, and Glx/tCr—plotted along the same mode. Curves show the mean across all Geneva study participants; shaded bands indicate variability estimated from the upper/lower bin envelopes (see Methods). Ratios were computed from water-referenced, CSF-corrected metabolite maps.

Supplementary Tables

Supplementary Table 1: Stability and consistency of MetSiM across perturbations. Pearson r for (i) individual vs. group MetSiM, (ii) edge-weight dispersion (T-test), and (iii) leave-one-metabolite-out, at $K_{\text{pert}} \in \{1, 50, 100\}$. Correlations were Fisher z–aggregated across subjects; two-sided p-values were computed and BH–FDR corrected.

K_{pert}	Analysis type	Comparison	r	SD	p-value
1	Individual vs Sample Mean	Edge weights	0.51	0.15	0.001
	Edge weights dispersion	T-Test	0.165	0.088	0.001
	Edge Weights Leave-Out	NAANAAG	0.32	0.07	0.001
		Ins	0.40	0.08	0.001
		GPCPCh	0.32	0.06	0.001
		GluGln	0.37	0.09	0.001
		CrPCr	0.40	0.08	0.001
50	Individual vs Sample Mean	Edge weights	0.60	0.17	0.001
	Edge weights dispersion	T-Test	0.095	0.050	0.001
	Edge Weights Leave-Out	NAANAAG	0.80	0.18	0.001
		Ins	0.96	0.22	0.001
		GPCPCh	0.81	0.22	0.001
		GluGln	0.88	0.21	0.001
		CrPCr	0.96	0.19	0.001
100	Individual vs Sample Mean	Edge weights	0.60	0.17	0.001
	Edge weights dispersion	T-Test	0.095	0.050	0.001
	Edge Weights Leave-Out	NAANAAG	0.81	0.18	0.001
		Ins	0.97	0.24	0.001
		GPCPCh	0.81	0.23	0.001
		GluGln	0.89	0.22	0.001
		CrPCr	0.97	0.20	0.001

Supplementary Table 2: Pearson correlations (r) between the empirical Geneva Study average MetSiM matrix and RandGeom baselines with/without gray-matter adjacency (GMAdj), for hemispheres (LH, RH) and combined (LH+RH). p-values are from the standard *two-sided* test of zero correlation.

Model	Correlation	p-value
RandGeom LH+RH	0.0821071884398875	$p < 10^{-5}$
RandGeom GMAdj LH+RH	0.1334061281041330	$p < 10^{-5}$
RandGeom LH	0.1143208517795050	$p < 10^{-5}$
RandGeom GMAdj LH	0.2087585798145300	$p < 10^{-5}$
RandGeom RH	0.1312569397124850	$p < 10^{-5}$
RandGeom GMAdj RH	0.2236959333642470	$p < 10^{-5}$

Supplementary Table 3: Optimal-path metrics for cortex and subcortex across modalities and hemispheres. The optimal path minimizes $\mathcal{L}(\gamma) = \nabla\mu(\gamma)/H(\gamma)$. lh/rh denote hemispheres.

		Cortex			Subcortex		
Modality	Hemis.	$\mathcal{L}(\gamma)$	$\nabla\mu(\gamma)$	$H(\gamma)$	$\mathcal{L}(\gamma)$	$\nabla\mu(\gamma)$	$H(\gamma)$
metabolic	lh	0.1048732	0.0813113	0.7753295	0.1735698	0.1296689	0.7470705
metabolic	rh	0.0978829	0.0681034	0.6957640	0.1855009	0.1389642	0.7491297
connectopy	lh	0.2530858	0.1601229	0.6326825	1.5494823	0.6095769	0.3934068
connectopy	rh	0.1432343	0.0923133	0.6444914	0.3833849	0.2046281	0.5337407
BigBrain	lh	0.0848387	0.0719600	0.8481973	N/A	N/A	–
BigBrain	rh	0.1259962	0.1043692	0.8283523	N/A	N/A	–
VonEconomo	lh	0.1419817	0.1204285	0.8481973	N/A	N/A	–
VonEconomo	rh	0.1440392	0.1221737	0.8481973	N/A	N/A	–
genotopy	lh	0.0760121	0.0667762	0.8784946	N/A	N/A	–
genotopy	rh	0.0852698	0.0706309	0.8283229	N/A	N/A	–

Supplementary Table 4: Overlap proportions between MetSiM-derived metabolic clusters ($K = 9$) and cyto.itectonic classes (Cognitive–Consilience dataset). The label correspondence was optimized with the Hungarian (Kuhn–Munkres) algorithm.

	cyto._1	cyto._2	cyto._3	cyto._4	cyto._5	cyto._6	cyto._7	cyto._8	cyto._9
metabolic_1	0.000	0.636	0.182	0.000	0.000	0.000	0.000	0.000	0.182
metabolic_2	0.261	0.261	0.174	0.043	0.174	0.087	0.000	0.000	0.000
metabolic_3	0.000	0.000	0.000	0.000	0.000	0.160	0.080	0.760	0.000
metabolic_4	0.080	0.080	0.440	0.360	0.040	0.000	0.000	0.000	0.000
metabolic_5	0.043	0.348	0.087	0.348	0.087	0.087	0.000	0.000	0.000
metabolic_6	0.000	0.448	0.448	0.000	0.103	0.000	0.000	0.000	0.000
metabolic_7	0.300	0.367	0.200	0.067	0.067	0.000	0.000	0.000	0.000
metabolic_8	0.000	0.200	0.100	0.100	0.000	0.100	0.200	0.150	0.150
metabolic_9	0.143	0.714	0.000	0.000	0.000	0.000	0.000	0.071	0.071

Supplementary Table 5: Complete details on MRSI acquisition, reconstruction, quantification, and validation are provided in accordance with the MRSinMRS checklist standard [7].

**Category**	**Geneva Study**	**Lausanne Psychosis Cohort**
Scanner	3T Magnetom TrioTim (Siemens)	3T Prisma Fit (Siemens)
RF coils	32 ch ^{1}H head coil	32 ch ^{1}H head coil
Coil elements	HEA; HEP	HEA; HEP
Sequence	3D ^{1}H–FID–MRSI (CS-accelerated)	3D ^{1}H–FID–MRSI (CS-accelerated)
Position	R4.8 A11.8 H36.9	L3.9 A23.0 H4.3
Orientation	T > C−13.5 > S2.5	T > C34.5 > S−5.4
Rotation (deg)	−2	0
TE (ms)	1.5	1
TR (ms)	372	353
Averages	1	1
Flip angle (°)	35	40
FOV (mm)	210 × 160 × 105	210 × 160 × 95
Slab thickness (mm)	95	95
Slabs	1	1
Resolution (mm^3)	5 × 5 × 5.3	5 × 5 × 5.3
Spectral bandwidth (Hz)	2000	2000
FID points / Vector size	512	512
Acquisition duration (ms)	256	256
Matrix size	32 × 42 × 20	32 × 42 × 20
Water reference TE (ms)	1.5	1.07
Water reference TR (ms)	36	25
Water reference flip angle (°)	3	5
Water reference resolution (mm^3)	6.6 × 6.7 × 6.6	6.6 × 6.7 × 6.6
Water reference FID points	16	16
Averaging mode	Short term	Short term
Water suppr.	Weak water suppr.	Water sat.
Water suppr. BW (Hz)	60	60
Spectral suppr.	None	None
Measurements	1	1
Saturation bands	2 bands, 20 mm thickness	2 bands, 20 mm thickness
Compressed sensing	acceleration factor 3.3, radius 0.2	acceleration factor 3.3, radius 0.2
Preparation scans	4	4
Dimension	3D	3D
Delta frequency (ppm)	0	0
Phase encoding	3D Sparse Elliptical Encoding	Elliptical
Remove oversampling	On	On
WS timing (ms)	24	22
WS amplitude factor	0.9	0.9
Max gradient amplitude used (mT/m)	26	33
Shim mode	Advanced	Advanced

Continued on next page

**Category**	**Geneva Study**	**Lausanne Psychosis Cohort**
Data processing	Low-rank + TGV reconstruction<br>Lipid/water removal	Low-rank + TGV reconstruction<br>Lipid/water removal
Quantification	LCModel	LCModel
Metabolite basis set (LCModel)	NAA, NAAG, Cr, PCr, GPC, PCh<br>mI , sI, Glu, Gln, Lac, GABA, GSH<br>Tau, Asp, Ala	NAA, NAAG, Cr, PCr, GPC, PCh<br>mI , sI, Glu, Gln, Lac, GABA, GSH<br>Tau, Asp, Ala
Combined metabolites	tNAA (NAA+NAAG), tCr (Cr+PCr)<br>Cho (GPC+PCh), Ins (mI)<br>Glx (Glu+Gln)	tNAA (NAA+NAAG), tCr (Cr+PCr)<br>Cho (GPC+PCh), Ins (mI)<br>Glx (Glu+Gln)
Quality metrics	SNR, CRLB (per metabolite), FWHM	SNR, CRLB (per metabolite), FWHM

References

[1] Alexander Schaefer, Ru Kong, Evan M Gordon, Timothy O Laumann, Xi-Nian Zuo, Avram J Holmes, Simon B Eickhoff, and BT Thomas Yeo. Local-global parcellation of the human cerebral cortex from intrinsic functional connectivity mri. *Cerebral cortex*, 28(9):3095–3114, 2018.

[2] Sebastian Urchs, Jonathan Armoza, Clara Moreau, Yassine Benhajali, Jolène St-Aubin, Pierre Orban, and Pierre Bellec. Mist: A multi-resolution parcellation of functional brain networks. *MNI Open Research*, 1:3, 2019.

[3] Benjamin A Thomas, Kjell Erlandsson, Marc Modat, Lennart Thurfjell, Rik Vandenberghe, Sebastien Ourselin, and Brian F Hutton. The importance of appropriate partial volume correction for pet quantification in alzheimer's disease. *European journal of nuclear medicine and molecular imaging*, 38:1104–1119, 2011.

[4] Andrew A Maudsley, C Domenig, V Govind, A Darkazanli, C Studholme, K Arheart, and C Bloomer. Mapping of brain metabolite distributions by volumetric proton mr spectroscopic imaging (mrsi). *Magnetic Resonance in Medicine: An Official Journal of the International Society for Magnetic Resonance in Medicine*, 61(3):548–559, 2009.

[5] Mohammed Z Goryawala, Sulaiman Sheriff, and Andrew A Maudsley. Regional distributions of brain glutamate and glutamine in normal subjects. *NMR in Biomedicine*, 29(8):1108–1116, 2016.

[6] Edgar Céléreau, Federico Lucchetti, Yasser Aleman, Daniella Dwir, Martine Cleusix, Jean-Baptiste Ledoux, Raoul Jenni, Meritxell Bach Cuadra, Zoé Schilliger, Alessandra Solida, et al. High-resolution whole-brain magnetic resonance spectroscopic imaging in youth at risk for psychosis. *bioRxiv*, pages 2025–06, 2025.

[7] Alexander Lin, Ovidiu Andronesi, Wolfgang Bogner, In-Young Choi, Eduardo Coello, Cristina Cudalbu, Christoph Juchem, Graham J Kemp, Roland Kreis, Martin Krššák, et al. Minimum reporting standards for in vivo magnetic resonance spectroscopy (mrsinmrs): experts' consensus recommendations. *NMR in Biomedicine*, 34(5):e4484, 2021.
