# Supplementary material for: Constructing the human brain metabolic connectome with MR spectroscopic imaging reveals cerebral biochemical organization
Source: Nat Commun. 2025 Dec 22;16:11344. doi: 10.1038/s41467-025-66124-w (PMC12727723; doi:10.1038/s41467-025-66124-w)
Supplement: Supplementary file 2 — Description of Additional Supplementary Files [file 41467_2025_66124_MOESM2_ESM.pdf]

## Description of Additional Supplementary Files

**Supplementary Data 1**. — MRSI brain coverage

**Supplementary Data 2**. — Results of the leave-one-GO-term-out, genes ranked as a function of their contribution to the correlation between metabolic similarity and genetic co-expression.

**Supplementary Data 3.** — Results of the leave-one-GO-term-out, genes ranked as a function of their contribution to the correlation between metabolic similarity and genetic co-expression and clustered with their respective Parent GO term.
