# Supplementary material for: Constructing the human brain metabolic connectome with MR spectroscopic imaging reveals cerebral biochemical organization
Source: Nat Commun. 2025 Dec 22;16:11344. doi: 10.1038/s41467-025-66124-w (PMC12727723; doi:10.1038/s41467-025-66124-w)
Supplement: Supplementary file 6 — Reporting Summary [file 41467_2025_66124_MOESM6_ESM.pdf]

nature portfolio

Corresponding author(s): Federico Lucchetti, Paul Klauser

Last updated by author(s): Oct 28, 2025

Reporting Summary

Nature Portfolio wishes to improve the reproducibility of the work that we publish. This form provides structure for consistency and transparency in reporting. For further information on Nature Portfolio policies, see our Editorial Policies and the Editorial Policy Checklist.

Statistics

For all statistical analyses, confirm that the following items are present in the figure legend, table legend, main text, or Methods section.

n/a	Confirmed	
☐	☒	The exact sample size (n) for each experimental group/condition, given as a discrete number and unit of measurement
☐	☒	A statement on whether measurements were taken from distinct samples or whether the same sample was measured repeatedly
☐	☒	The statistical test(s) used AND whether they are one- or two-sided<br>*Only common tests should be described solely by name; describe more complex techniques in the Methods section.*
☒	☐	A description of all covariates tested
☐	☒	A description of any assumptions or corrections, such as tests of normality and adjustment for multiple comparisons
☐	☒	A full description of the statistical parameters including central tendency (e.g. means) or other basic estimates (e.g. regression coefficient) AND variation (e.g. standard deviation) or associated estimates of uncertainty (e.g. confidence intervals)
☐	☒	For null hypothesis testing, the test statistic (e.g. F, t, r) with confidence intervals, effect sizes, degrees of freedom and P value noted<br>*Give P values as exact values whenever suitable.*
☒	☐	For Bayesian analysis, information on the choice of priors and Markov chain Monte Carlo settings
☒	☐	For hierarchical and complex designs, identification of the appropriate level for tests and full reporting of outcomes
☐	☒	Estimates of effect sizes (e.g. Cohen's d, Pearson's r), indicating how they were calculated

Our web collection on statistics for biologists contains articles on many of the points above.

Software and code

Policy information about availability of computer code

Data collection	The analysis code as well as third party software packages used in this study is available at GitHub: https://github.com/MRSI-Psychosis-UP/MRSI-Metabolic-Connectome
Data analysis	The analysis code as well as third party software packages used in this study is available at GitHub: https://github.com/MRSI-Psychosis-UP/MRSI-Metabolic-Connectome

For manuscripts utilizing custom algorithms or software that are central to the research but not yet described in published literature, software must be made available to editors and reviewers. We strongly encourage code deposition in a community repository (e.g. GitHub). See the Nature Portfolio guidelines for submitting code & software for further information.

Data

Policy information about availability of data

All manuscripts must include a data availability statement. This statement should provide the following information, where applicable:

- Accession codes, unique identifiers, or web links for publicly available datasets
- A description of any restrictions on data availability
- For clinical datasets or third party data, please ensure that the statement adheres to our policy

The processed derivative data (parcellation masks, within subject and group-level metabolic similarity matrices, figure source data, and analysis scripts) are available at https://github.com/MRSI-Psychosis-UP/MRSI-Metabolic-Connectome. Source data underlying the main figures are provided with this paper in the accompanying Source Data files.

Research involving human participants, their data, or biological material

Policy information about studies with human participants or human data. See also policy information about sex, gender (identity/presentation), and sexual orientation and race, ethnicity and racism.

Reporting on sex and gender	Sex of volunteers have been recorded but have not been investigated in this study. Gender of volunteers have not been recorded in this study.
Reporting on race, ethnicity, or other socially relevant groupings	Race, ethnicity or other socially relevant grouping of volunteers have been recorded but have not been investigated in this study.
Population characteristics	N/A
Recruitment	Healthy volunteers have been recruited from the general population.<br>Geneva study: All participants (assent) and their legal guardians (consent)<br>provided written informed consent/assent in accordance with the Geneva Regional Ethics Committee.<br>No a priori sex- or gender-based analyses were planned given the study's scope and sample size.<br>Lausanne cohort: Written informed consent was obtained from adult participants; for<br>minors, written parental/guardian consent and participant assent were obtained. The study protocol was<br>approved by the Cantonal Ethics Committee for Research on Human Beings (PB 2017-00675 82/14).
Ethics oversight	This research complies with all relevant ethical regulations. This study is a secondary analysis of deidentified MRI/MRSI data from two independent cohorts that were conducted previously and approved<br>by their respective ethics committees: the Geneva Cantonal Research Ethics Committee (CCER; protocol 2018-01731) and the Cantonal Research Ethics Commission on Human Research, Canton of Vaud,<br>Switzerland (CER-VD; protocol PB 2017-00675 82/14). Data handling complied with applicable dataprotection laws and institutional policies; only de-identified data were used and no re-identification was<br>attempted

Note that full information on the approval of the study protocol must also be provided in the manuscript.

Field-specific reporting

Please select the one below that is the best fit for your research. If you are not sure, read the appropriate sections before making your selection.

☒ Life sciences ☐ Behavioural & social sciences ☐ Ecological, evolutionary & environmental sciences

For a reference copy of the document with all sections, see nature.com/documents/nr-reporting-summary-flat.pdf

Life sciences study design

All studies must disclose on these points even when the disclosure is negative.

Sample size	n=69 for the Geneva study and n=13 for the Lausanne cohort.
Data exclusions	MRI data was excluded based on visual inspection of image quality. For the Geneva study cohort Exclusion criteria included chronic somatic diseases or significant medical conditions, recent psychotherapy (< 6 months), recent use of psychotropic medications (< 6 months), and any history of psychiatric disorders, except for current anxiety disorders or a past episode of major depressive<br>disorder resolved at least 6 months earlier.<br>For the Lausanne cohort volunteers were assessed by the Diagnostic Interview for<br>Genetic Studies [Presig et. al] to exclude a major mood, psychotic, or substance use disorder or had a first-degree<br>relative with a psychotic disorder.<br><br>Martin Preisig, Brenda T Fenton, Marie-Louise Matthey, Alexandre Berney, and François Ferrero.<br>Diagnostic interview for genetic studies (digs): inter-rater and test-retest reliability of the french<br>version. European archives of psychiatry and clinical neuroscience, 249:174–179, 1999.

Replication	Construction of metabolic similarity matrices was replicated on a independent cohort (Lausanne cohort) scanned on a different site.
Randomization	N/A
Blinding	N/A

Reporting for specific materials, systems and methods

We require information from authors about some types of materials, experimental systems and methods used in many studies. Here, indicate whether each material, system or method listed is relevant to your study. If you are not sure if a list item applies to your research, read the appropriate section before selecting a response.

Materials & experimental systems

n/a	Involved in the study
☒	☐ Antibodies
☒	☐ Eukaryotic cell lines
☒	☐ Palaeontology and archaeology
☒	☐ Animals and other organisms
☐	☒ Clinical data
☒	☐ Dual use research of concern
☒	☐ Plants

Methods

n/a	Involved in the study
☒	☐ ChIP-seq
☒	☐ Flow cytometry
☐	☒ MRI-based neuroimaging

Clinical data

Policy information about clinical studies

All manuscripts should comply with the ICMJE guidelines for publication of clinical research and a completed CONSORT checklist must be included with all submissions.

Clinical trial registration	We did not recruit participants for this study but relied on MRI data obtained in the context of two independent studies.
Study protocol	Study protocols for both cohorts have been published here:<br><br>Geneva study:<br>Piguet C, Klauser P, Celen Z, James Murray R, Magnus Smith M, Merglen A. Randomized controlled trial of a mindfulness-based intervention in adolescents from<br>the general population: The Mindfulteen neuroimaging study protocol. Early<br>Intervention in Psychiatry. 2022;16(8):891–901.<br><br>Lausanne cohort:<br>Baumann, P. S., Crespi, S., Marion-Veyron, R., Solida, A., Thonney, J., Favrod, J., Bonsack,<br>C., Do, K. Q., & Conus, P. (2013). Treatment and early intervention in psychosis<br>program (TIPP-Lausanne): Implementation of an early intervention programme for<br>psychosis in Switzerland. Early Interv Psychiatry, 7(3), 322-328.<br>doi:10.1111/eip.12037
Data collection	See "Study Protocol"
Outcomes	See "Study Protocol"

Plants

Seed stocks	N/A
Novel plant genotypes	N/A
Authentication	N/A

Magnetic resonance imaging

Experimental design

Design type	Resting state
Design specifications	3 MRI sequences acquired in a time interval of 40 minutes
Behavioral performance measures	N/A

Acquisition

Imaging type(s)	T1 weighted, Diffusion weighted, whole brain MRSI
Field strength	3T

Sequence & imaging parameters

For the Geneva study, magnetic resonance (MR) data were acquired using a 3-Tesla scanner (Magnetom TrioTim, Siemens Healthineers, Forchheim, Germany) equipped with a 32-channel head coil at the Brain and Behavior Laboratory in Geneva. Each scanning session included a magnetization-prepared rapid acquisition gradient echo (MPRAGE) T1-weighted sequence with an in-plane resolution of 1 mm and a slice thickness of 1.2 mm, covering a volume of 240 × 257 × 160 voxels. The repetition time (TR), echo time (TE), and inversion time (TI) were set to 2300 ms, 2.98 ms, and 900 ms, respectively. Additionally, a diffusion spectrum imaging (DSI) sequence was performed, acquiring 128 diffusion-weighted images with a maximum b-value of 8000 s/mm2 and one b0 reference image. The acquisition volume for DSI consisted of 96 × 96 × 34 voxels with a resolution of 2.2 × 2.2 × 2.2 mm. The TR and TE for the DSI sequence were 6800 ms and 144 ms, respectively.

For the Lausanne Psychosis Cohort, MRI sessions were conducted on a 3-Tesla scanner (MAGNETOM Prisma fit, Siemens Healthineers, Forchheim, Germany) at the Lausanne University Hospital for 13 healthy controls. While the MPRAGE and DSI sequences parameters were identical to those used in the Mindfulteen study, the the 3D 1H-FID-MRSI sequence had different parameters than the one implemented for the Geneva study (TE = 1.0 ms, TR = 353 ms and flip angle of 40 deg. The FoV was the same as in Lausanne, with the same spatial resolution of 5×5 × 5.3 mm, as the spectral bandwidth and FID size. The water acquisition had the following parameters: same TE of 1.07 ms, TR of 25 ms, and a flip angle of 5 deg. FOV size, resolution, bandwidth and vector size were the same as for the Geneva study.
Complete details on MRSI acquisition, reconstruction, quantification, and validation are provided in the original methodological reference Klauser et al. (2022),
while the MRSI parameters are reported in the MRSinMRS checklist (Supplementary Data) Lin et al. (2021).
The 3D 1H-FID-MRSI sequence accelerated by compressed-sensing Klauser et al. (2022) was acquired with 1.50 ms TE, 372 ms TR and 35 deg flip angle. The Fieldof-View (FoV) size was 210 × 160 × 105 mm (anterior-posterior, right-left, head-foot directions) with a 95 mm-thick slab selection, with a spatial resolution of 5×5 × 5.3 mm and the spectral bandwidth was 2 kHz acquired with 512 points. The reference water acquisition had the following parameters: same TE, TR of 25 ms, lower flip angle of 3°, same FOV size, lower resolution of 6.6 x 6.7 x 6.6 mm, same bandwidth and free induction decay (FID) size of 16 points.

Klauser A, Klauser P, Grouiller F, Courvoisier S, Lazeyras F. Whole-brain highresolution metabolite mapping with 3D compressed-sensing SENSE low-rank 1H
FID-MRSI. NMR in Biomedicine. 2022;35(1):e4615.

Area of acquisition: *State whether a whole brain scan was used OR define the area of acquisition, describing how the region was determined.*

Diffusion MRI ☒ Used ☐ Not used

Parameters: The diffusion spectrum imaging (DSI) sequence was performed, acquiring 128 diffusion-weighted images with a maximum b-value of 8000 s/mm2 and one b0 reference image. The acquisition volume for DSI consisted of 96 × 96 × 34 voxels with a resolution of 2.2 × 2.2 × 2.2 mm. The TR and TE for the DSI sequence were 6800 ms and 144 ms, respectively

Preprocessing

Preprocessing software	For each subject, the anatomical T1-weighted image was used to parcellate the brain

into different regions using the FreeSurfer package (version 7.2.0, http://surfer.nmr.
mgh.harvard.edu) and subsequently identify nine distinct supra-regions: cortex, basal
ganglia, thalamus, amygdala, hippocampus, hypothalamus, cerebellum, brainstem,
and white matter. We relied on the Chimera parcellation software tool Aleman-Gomez (2024) to create a combined volumetric parcellation for each input subject anatomical image based
on the following atlases (as listed in the original Chimera repository) and referred to
as the LFMIHIFS-3 parcellation scheme.
The MRSI data was reconstructed using a low-rank model constrained by totalgeneralized variation, with prior removal of subcutaneous lipid contamination and
residual water signals Klauser et al. (2021). Following reconstruction, the spatiospectral data was analyzed using LCModel Provencher (2001) to quantify metabolite
signal in each voxel, using the water signal from the additional acquisition as a reference. Because of the ultra-short echo time (1.5 ms), no T2 relaxation corrections were
required Klauser et al. (2021). Although T1 weighting is present because of the reduced
TR and would differentially affect GM and WM, only GM–GM correlations were considered in this study. Thus, explicit T1 correction would merely introduce a global
scaling factor without altering the reported results. However, the 5 mm isotropic resolution may introduce partial signal leakage across GM–WM boundaries; therefore,
potential GM–WM composition effects were further mitigated using voxel-wise partial volume correction in combination with a MRI point-spread-function correction
(see Methods Section 4.2.5).
The basis set for LCModel fitting included the following metabolites: Nacetylaspartate (NAA), N-acetylaspartylglutamate (NAAG), creatine (Cr), phosphocreatine (PCr), glycerophosphocholine (GPC), phosphocholine (PCh), myo-inositol
(mI), scyllo-inositol (sI), glutamate (Glu), glutamine (Gln), lactate (Lac), gammaaminobutyric acid (GABA), glutathione (GSH), taurine (Tau), aspartate (Asp), and
alanine (Ala). Only a subset of these metabolites could be reliably resolved and due
to overlapping spectral peaks, certain metabolites were combined: NAA and NAAG
(denoted tNAA), Cr and PCr (denoted tCr), GPC and PCh (denoted Cho), mI
(denoted Ins), and Glu and Gln (denoted Glx), resulting in five distinct metabolite
volumes. LCModel also provided spectral quality metrics, such as the signal-to-noise
ratio (SNR), Cramer-Rao Lower Bound (CRLB) for each metabolite estimation.

Normalization: The MRSI tCr volume was co-registered to the corresponding T1-weighted anatomical image using Advanced Normalization Tools (ANTs) Tustison et al. (2021), with
mutual information as the cost function for optimization. In addition, each individual
T1-weighted image was nonlinearly registered to the MNI152 standard space. Both
registration steps yielded a combination of affine and symmetric image normalization
(SyN) transforms, which were subsequently applied or inverted, as appropriate, in
later preprocessing and parcellation steps.

Normalization template: N/A

Noise and artifact removal: N/A

Volume censoring: Low CRLB values, returned by the LC model.

Statistical modeling & inference

Model type and settings: N/A

Effect(s) tested: N/A

Specify type of analysis: [x] Whole brain [ ] ROI-based [ ] Both

Statistic type for inference: N/A

(See Eklund et al. 2016)

Correction: N/A

Models & analysis

n/a	Involved in the study
[ ]	[ ] Functional and/or effective connectivity
[ ]	[x] Graph analysis
[ ]	[x] Multivariate modeling or predictive analysis

Functional and/or effective connectivity: N/A

Graph analysis: Network graphs were generated by binarizing weighted matrices (similarity or connectivity) based on a specified edge density p. For matrices containing negative values,

negative weights were rectified to their absolute values before applying the binarization threshold. This threshold was chosen to achieve the desired proportion of binarized connections ρ. Graph analyses were conducted across a wide range of edge densities (1 to 30 %) for each weighted matrix.
We computed the rich-club coefficient ϕ(k) for a given binarized network. We calculated the centrality of a node using degree centrality, which measures the number of direct connections a node has to other nodes in the network.
For each binarized graph, we assessed the statistical significance of the observed network metrics by generating an ensemble of 1,000 randomized networks using the Maslov-Sneppen rewiring procedure, which preserves the number of nodes, total number of edges, and the degree distribution of the original graph while randomizing the specific wiring of edges. We then computed empirical p-values for each topological metric by comparing the observed value to the distribution of corresponding values across the 1,000 random graphs. Specifically, for each metric, the p-value was calculated as the fraction of randomized networks in which the metric was greater than or equal to the observed value.
The higher-order connectivity of a weighted or binarized network matrix A was computed as the k-th order cosine similarity of A with itself

Multivariate modeling and predictive analysis

To compute the influence of distant nodes from a structural connectivity matrix S on the state of the MetSiM M, we relied on a communicabilty model Kondor and Lafferty (2002) and estimated the Green's function of the network heat equation , which corresponds to the communicability expression.
